# Statin-Associated Necrotizing Myopathy: A Feared Complication

**DOI:** 10.7759/cureus.11689

**Published:** 2020-11-24

**Authors:** Suong Nguyen, Swetha Ann Alexander, Sandra Apenteng, Andrew Castiglione

**Affiliations:** 1 Internal Medicine, University of Connecticut, Farmington, USA; 2 Internal Medicine, Hartford Hospital, Hartford, USA

**Keywords:** statin, statin-induced, myopathy, rhabdomyolysis, weakness, hmg-coa reductase

## Abstract

Statins are a group of frequently-prescribed drugs with proven cardiovascular risk-benefit. The most common adverse effects include weakness and myalgias. However, prescribers need to be aware of a less common complication, statin-associated necrotizing myopathy, which can occur at any time during the treatment course and has been found to be <0.1% of adverse effects. High suspicion is warranted when patients taking statins develop weakness and myalgia. Increased risk of muscle injury has been observed when using gemfibrozil in combination with statins and should be avoided. We present a case of an elderly male with chronic use of combination lipid-lowering agents who initially presented with proximal weakness. He was diagnosed with statin-associated necrotizing myopathy and subsequently developed rapid end-stage renal disease in the setting of severe rhabdomyolysis. The case report discusses the work-up of proximal muscle weakness with focus on the importance of early recognition and prompt management of rhabdomyolysis to avoid life-threatening complications.

## Introduction

Since the 1980s, statins (hydroxymethylglutaryl-CoA reductase inhibitors) have undergone remarkable changes from semi-synthetic to synthetic formulations, with atorvastatin being the most commonly prescribed [1-3]. Large clinical trials have proven primary and secondary prevention of cardiovascular events contributing to its widespread use and integration into clinical practice guidelines [4]. Among the very same cohorts that showed significant cardiovascular benefits, adverse effects ranging from myalgia to necrotic myopathy have been reported. Statin-induced rhabdomyolysis occurs in 0.44 per 10,000 patients a year, with incidence increasing to 5.98 per 10,000 patients when combined with fibrates [3,5].

We describe a case of an elderly man presenting with proximal limb weakness diagnosed with statin-associated necrotizing myopathy in the setting of decompensated renal function. We aim to discuss the workup of proximal muscle weakness and current treatment regimens for severe cases of rhabdomyolysis. 

## Case presentation

A 74-year-old male with a history of atrial fibrillation, heart failure with reduced ejection fraction (HFrEF), hypertension, hyperlipidemia, insulin-dependent diabetes, active diverticulitis on antibiotics, and chronic kidney disease (CKD) stage III (baseline S.cr 2-2.5 mg/dL) presented with two weeks of worsening fatigue and weakness with a 20 lb weight loss. He was hemodynamically stable, alert and oriented, saturating at 92% on room air with normal lung sounds. Symmetrical proximal muscle weakness of the bilateral upper and lower extremities was present with failure to resist force at hip flexors. Distal strength, sensation, and reflexes were intact and symmetric bilaterally and no rashes were noted. Metabolic profile indicated anion gap metabolic acidosis and acute kidney injury with serum creatinine of 5.3 mg/dL, blood urea nitrogen (BUN) of 78 mg/dL, bicarbonate of 15 mmol/L and normokalemia with lactic acid of 1.2 mmol/L, creatinine kinase (CK) levels of 9,951 U/L (24-204 U/L) and urinalysis with proteinuria and myoglobinuria. Medication reconciliation revealed current intake of rosuvastatin 40 mg daily (previously on high-dose atorvastatin) and gemfibrozil 600 mg twice daily.

**Figure 1:**
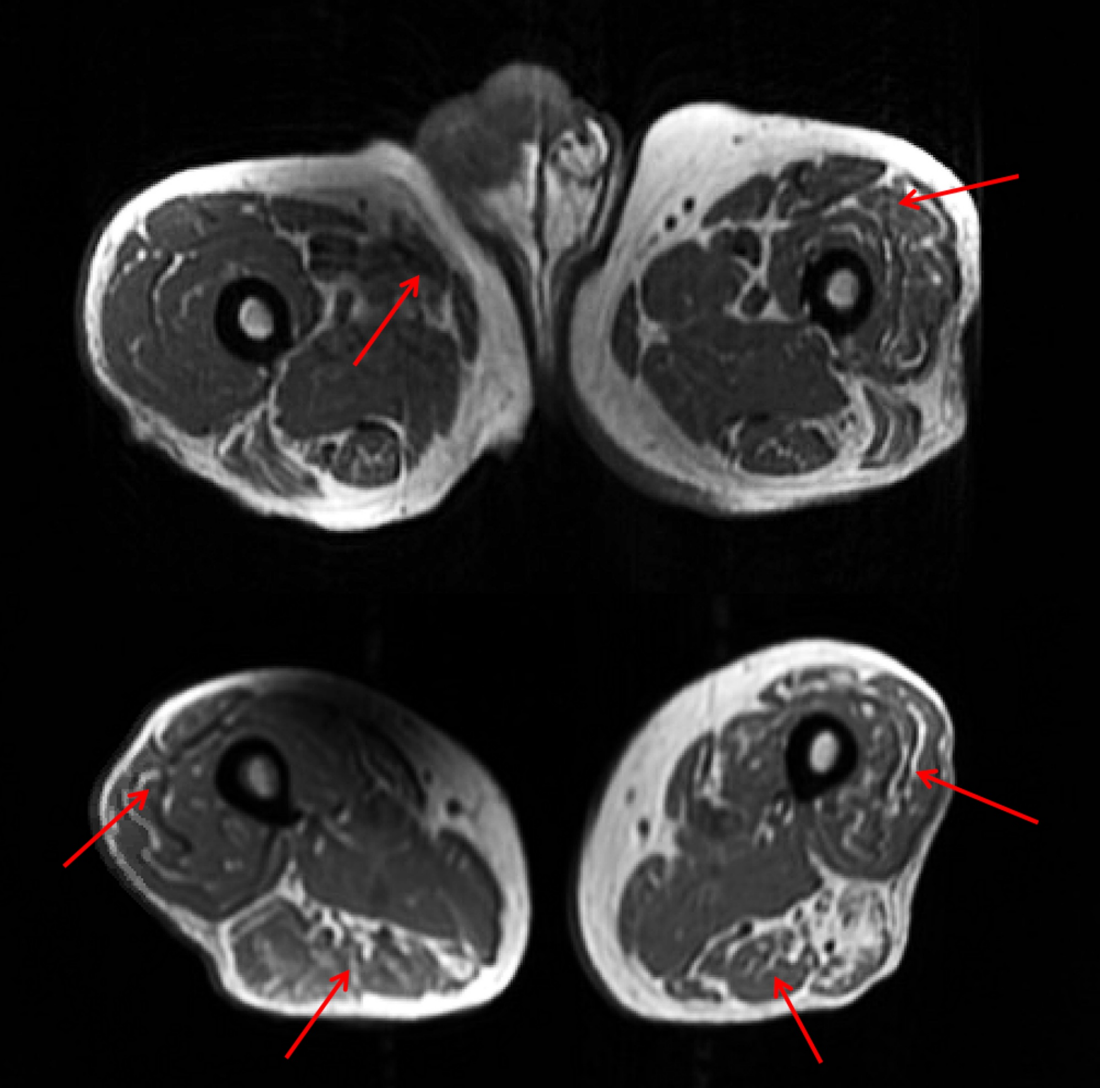
Axial T1 fast spin-echo (FSE) of the upper and lower bilateral thighs with mild edema diffusely in the anterior muscles of the proximal left and right thigh (top) with increased hyperintensity in the mid to distal thigh muscles bilaterally suggestive of muscular edema (bottom).

MRI revealed diffuse and patchy muscular edema of the bilateral thighs, seen in Figure 1. Further work-up demonstrated thyroid-stimulating hormone (TSH) of 1.10 mIU/L, c-reactive protein (CRP) of 1.55 U/L, erythrocyte sedimentation rate (ESR) of 114 MM/HR, antinuclear antibody (ANA) of 1:80, complement total (CH50) of >60 units/mL (31-60 U/mL), 3-hydroxy-3-methyl-glutaryl-coenzyme A reductase (HGMR) antibody, immunoglobulin G (IgG) <3 units (0-19 units) and negative myositis panel.

Despite statin discontinuation and aggressive intravenous hydration with lactated ringers, his symptoms progressed and CK levels continued to rise. Nephrology and Rheumatology services were consulted within 24 hours of admission for recommendations given high suspicion for statin-induced necrotizing myopathy. He was subsequently started on methylprednisone 60 mg twice daily and intravenous immunoglobulin (IVIG) within the same day. On day seven of admission, the patient became acutely encephalopathic in the setting of uremia and he was started on continuous renal replacement therapy for worsening anuric acute renal failure with acute tubular necrosis. Electromyography (EMG) was deferred due to severity of the course and left thigh muscle biopsy was performed following immunosuppressive treatment on day 16, which revealed marked myofiber necrosis and regeneration consistent with acute rhabdomyolysis. Unfortunately, the patient continued to deteriorate and suffered severe depression leading to decline of further treatment resulting in transition to comfort care on day 19 of admission. 

## Discussion

Before diagnosing a patient with statin-induced myopathy it is important to evaluate differential diagnoses with similar presentations. Proximal weakness is defined in terms of function. Patients with proximal weakness often face new onset difficulty performing tasks such as climbing stairs or combing hair. Given various causes of proximal weakness (Table 1) it is pertinent to obtain a thorough medical history, medication reconciliation, alcohol and drug history, and evaluation for toxins and metabolic disturbances. 

**Table 1:** Differential diagnosis of proximal weakness and characteristic features CK: creatinine kinase, EMG: electromyography, HMG-CoA: 3-hydroxy-3-methylglutaryl coenzyme A

Diagnosis	Characteristic Features
Inflammatory	
Polymyositis	Proximal muscle weakness with elevated CK and aldolase. EMG shows typical features of muscle involvement. Biopsy reveals cellular infiltrate within the fascicle with cytotoxic CD8+ T cells.
Dermatomyositis	Features similar to polymyositis with specific skin findings of Gottron’s papules or heliotrope rash. Biopsy reveals cellular infiltrate mainly in perifascicular area with ​B-lymphocytes.
Skeletal muscle vasculitis with chronic rheumatological conditions	Associated with a chronic inflammatory condition and may have systemic symptoms. Biopsy reveals fibrinoid necrosis of the vessel wall with transmural inflammation.
Endocrine	
Hypothyroidism/hyperthyroidism	Proximal muscle weakness with myoedema and abnormal thyroid-stimulating hormone levels with resolution occurring when euthyroid.
Cushing’s syndrome	Associated with proximal myalgias with normal EMG and CK and myoglobin levels. Biopsy reveals atrophy of muscles.
Electrolyte disorders	
Hypokalemia, hypophosphatemia, hypocalcemia, hypo/hypernatremia	Acute to subacute onset with cramp-like weakness with normal to elevated CK levels. EMG may show "myopathic" motor unit potentials with biopsy revealing myofiber vacuolation and necrosis.
Metabolic myopathy	
Carbohydrate, lipid or purine metabolic abnormality	Onset may be at young-age with exercise intolerance and cramping of proximal and distal muscles. Systemic involvement may present causing cardiomyopathy and neuropathy.
Drugs and toxins	
Alcohol	May present with acute or chronic rhabdomyolysis with biopsy revealing diffuse muscle atrophy.
Cocaine and heroine	May lead to non-traumatic rhabdomyolysis.
HMG-CoA reductase inhibitors (Others-penicillamine, zidovudine, colchicine, antimalarial drugs)	Persistently elevated CK levels with muscle histology revealing muscle necrosis with minimal inflammation.
Rhabdomyolysis	
Crush trauma, seizures, delirium tremens, extreme exertion, malignant hyperthermia	Markedly elevated CK levels with myalgia and myoglobinuria.
Inherited myopathies	
Muscular dystrophy	Elevated CK and transaminases at onset of birth and may further require genetic analysis.

Myopathy is a broad term used to describe muscular dysfunction with skeletal muscle weakness being a principal symptom. Indirect and direct muscle injury may lead to myopathies and in its most severe instances may present as rhabdomyolysis. Predisposing risk-factors include elderly age, female gender, renal insufficiency, hepatic dysfunction, hypothyroidism and polypharmacy. As mentioned prior, statins have been shown in numerous large cohort studies to increase the risk of rhabdomyolysis with an estimated 2.3 per 10,000 person-years [5]. Although all statins increase the risk of muscle injury by 0.1% to 0.5%, rosuvastatin at varying doses has been shown in studies to increase the risk of muscle involvement to 1% compared to other statins [5,6]. A small retrospective study involving 45 patients observed the mean duration of statin therapy of 6.3 months before onset of symptoms with a mean duration of myalgia of 2.3 months after statin discontinuation [7]. An increased risk of myopathy has also been observed between drug-drug interactions, particularly with fibrates, with reports of a 5.5-fold increase with statin-fibrate therapy compared to statin monotherapy through a mechanism that increases the overall plasma concentration of the used statin [5,7]. In doing so, 3-hydroxy-3-methylglutaryl coenzyme A (HMG-CoA) reductase becomes inhibited preventing conversion to mevalonate, a precursor for producing isoprenoids [3,8]. The lack of isoprenoids causes inactivation of GTPases preventing cell membrane anchorage altering cellular function causing decreased cholesterol concentrations in the cell membrane with subsequently impaired mitochondrial enzyme activity leading to muscle injury [3]. 

Rhabdomyolysis should be suspected in patients presenting with acute weakness, myalgias, acute renal failure, and myoglobinuria following investigation starting with routine lab work. In severe cases, a basic metabolic profile (BMP) may reveal acute renal dysfunction with metabolic acidosis secondary to lactic acidosis with electrolyte abnormalities such as hypocalcemia, hyperkalemia, and hyperphosphatemia [2,5,9]. Myonecrosis leads to protease release into the systemic circulation causing elevations of myoglobin, CK, lactate dehydrogenase, and hydroxybutyrate dehydrogenase, which lead to elevated transaminases reflecting hepatic dysfunction seen in up to 25% of patients with rhabdomyolysis [10]. Serum CK levels may be greater than 10 times the upper limit of normal with rise in two to 12 hours after onset of injury peaking at three to five days and declining in six to 10 days [3,7,9,11]. Urinalysis may reveal “tea colored urine” due to myoglobinuria. It is imperative to perform a medical reconciliation and drug toxicity screen to exclude illicit substances as potential contributors [9]. It is necessary to rule out autoimmune causes of myopathy by testing for myositis specific antibodies (MSA) including, but not limited to, anti-Sjögren’s type A (anti-Jo-1/SSA), anti-Sjögren’s type B (anti-La/SSB), and anti-signal recognition particle (anti-SRP) for polymyositis, dermatomyositis, systemic lupus erythematosus, and Sjögren’s syndrome [8,11]. In certain circumstances in which immunosuppressives are promptly started before MSA results are available, it may necessitate muscle biopsy to confirm the presence of perivascular atrophy and/or lymphocytic cellular infiltrates related to these autoimmune disorders [11]. Further testing for 3-hydroxy-3-methylglutaryl coenzyme A reductase (HGMR) antibody will evaluate for statin necrotizing myopathy and thyroid stimulating hormone (TSH) will determine presence of thyroid dysfunction.

In situations of worsening rhabdomyolysis further diagnostic studies are warranted. An MRI can be used to assess the extent of muscle involvement and determine the site of muscle biopsy, which is typically the thigh or biceps. In statin-associated myopathy, MRI demonstrates the presence of intramuscular fatty infiltration with or without edema with minimal inflammation [11,12]. Electromyography generally presents irritable myopathy and muscle biopsy reveals myofiber atrophy with necrosis without inflammation [12]. 

Statins and offending agents should be discontinued immediately when symptoms present. Treatment begins with volume resuscitation to expand the extracellular fluid compartment. Aggressive IV hydration should be administered early and continued until plasma CK levels decrease to 1,000 U/l or below [5,6]. Sodium bicarbonate may be used to alkalinize urine to decrease cast formation and minimize renal tubule injury secondary to the toxic effects of myoglobin. Due to lack of studies, there are no guidelines recommending fluid type and rate in regard to hydration in the setting of rhabdomyolysis.

Steroids, intravenous immunoglobulin (IVIG), and immunomodulating agents have become the mainstay treatment for statin-associated myopathy observed in the literature [8,12]. If symptoms continue to worsen despite statin discontinuation, then prompt initiation of steroids, often at the gestalt of the clinician providing care, is warranted. The use of prednisone 1-2mg/kg/day dosage or pulse dose steroids of methylprednisone 1g daily for three days in cases has been and has demonstrated a positive effect [8,12]. With failed clinical improvement in weeks to months with steroid administration, studies have demonstrated some efficacy of using immunomodulating agents such as methotrexate or rituximab as second line agents [8,12]. Further management with IVIG monotherapy is considered with doses of 2mg/kg divided over a two- to five-day course. IVIG has shown modest success in two different studies demonstrating at least a partial improvement in patient's symptoms by 36% and 54%, with complete to near-complete resolution of symptoms occurring 64% and 46% respectively [13,14]. It is unfortunate we will not know if our patient would have had resolution of his symptoms if treatment were to have continued. There is also a question if further immunomodulating agents would have had any effect on his illness.

## Conclusions

Statins are commonly prescribed medications in medical practice and should be discontinued with the first signs of statin intolerance. In patients presenting with proximal muscle weakness; inflammatory, infectious, toxic, endocrine, or metabolic causes should be thoroughly evaluated. Statin-associated necrotic myopathy may occur at any time after drug initiation and should be considered in the patient with muscle weakness irrespective of the time course of initiation and if left unconsidered may be fatal. Prompt and aggressive volume restoration is critical to prevent progression to acute renal failure and the need for renal replacement therapy. Steroids and immunomodulating treatment should be considered if symptoms fail to improve. However, there continues to be a need for randomized controlled trials to further study the treatment of statin-associated necrotic myopathy as patients continue to be treated based on symptom severity and clinical presentation relying on observations in retrospective studies and expert consensus.
